# Supplementary material for: Genome-Wide Transcriptome Analysis of Rice Seedlings after Seed Dressing with Paenibacillus yonginensis DCY84T and Silicon
Source: Int J Mol Sci. 2019 Nov 23;20(23):5883. doi: 10.3390/ijms20235883 (PMC6928808; doi:10.3390/ijms20235883)
Supplement: Supplementary file 1 [file ijms-20-05883-s001.zip › Figure S1.pptx]

## Slide 1
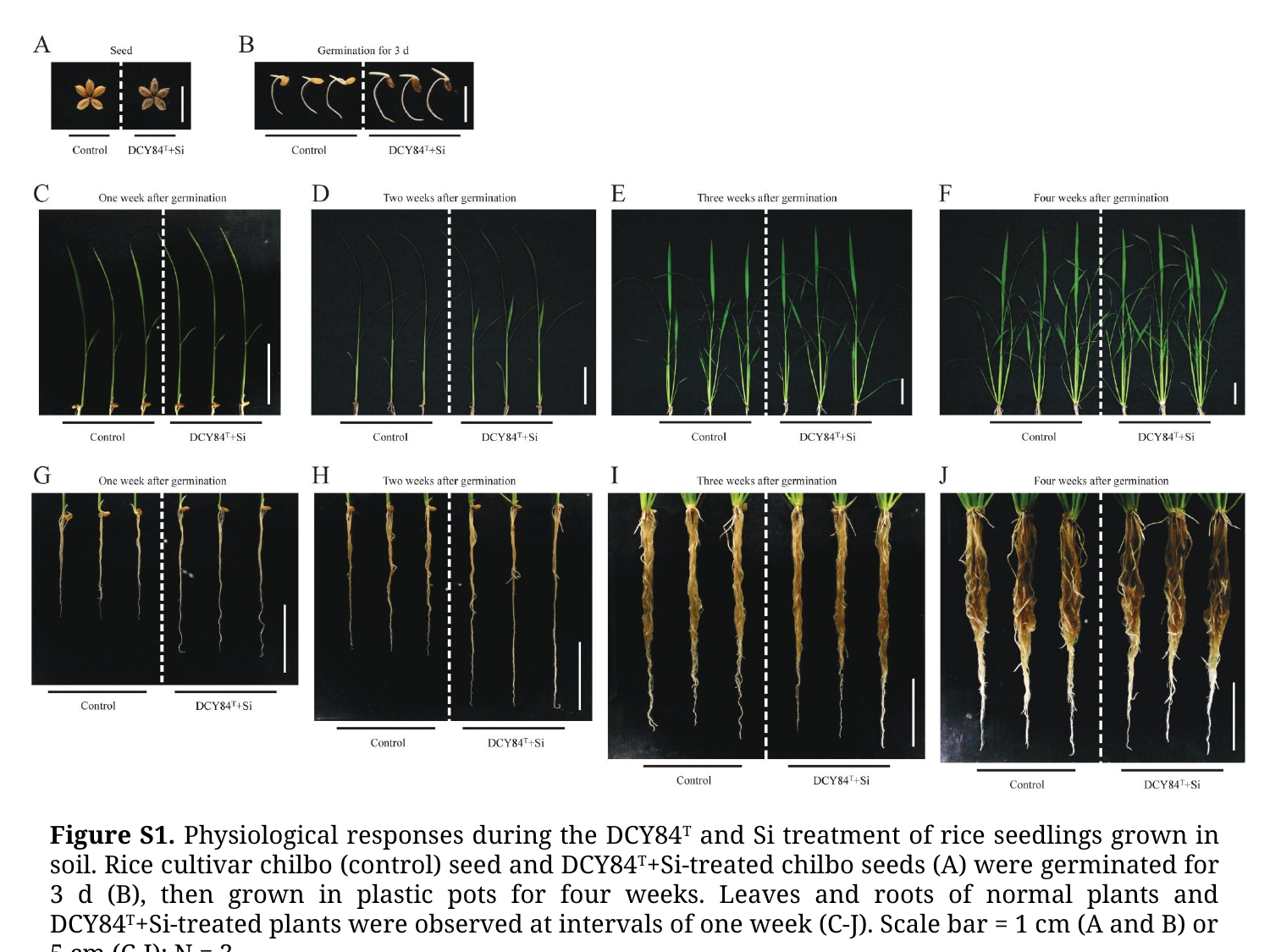

Figure S1. Physiological responses during the DCY84T and Si treatment of rice seedlings grown in soil. Rice cultivar chilbo (control) seed and DCY84T+Si-treated chilbo seeds (A) were germinated for 3 d (B), then grown in plastic pots for four weeks. Leaves and roots of normal plants and DCY84T+Si-treated plants were observed at intervals of one week (C-J). Scale bar = 1 cm (A and B) or 5 cm (C-J); N = 3.
